# Biomimetic dual sensing polymer nanocomposite for biomedical applications

**DOI:** 10.3389/fbioe.2024.1322753

**Published:** 2024-02-20

**Authors:** Abdalla M. Omar, Mohamed H. Hassan, Evangelos Daskalakis, Albert Smith, Jack Donoghue, Wajira Mirihanage, Paulo J. D. S. Bartolo

**Affiliations:** ^1^ Department of Mechanical, Aerospace, and Civil Engineering, University of Manchester, Manchester, United Kingdom; ^2^ Singapore Centre for 3D Printing (SC3DP), School of Mechanical and Aerospace Engineering, Nanyang Technological University, Singapore, Singapore; ^3^ TESCAN-UK Ltd., Cambridge, United Kingdom; ^4^ Department of Materials, The University of Manchester, Manchester, United Kingdom

**Keywords:** electrospinning, sensor, nanocomposites, PVDF, carbon black

## Abstract

There is a growing need for sensing materials that can provide multiple sensing capabilities for wearable devices, implantable sensors, and diagnostics tools. As complex human physiology requires materials that can simultaneously detect and respond to slow and fast pressure fluctuations. Mimicking the slow adaptive (SA) and fast adaptive (FA) mechanoreceptors in skin can lead to the development of dual sensing electrospun polymer nanocomposites for biomedical applications. These dual sensing nanocomposites can provide simultaneous sensing of both slow and fast pressure fluctuations, making them ideal for applications such as monitoring vital signs, detecting a wider range of movements and pressures. Here we develop a novel dual sensing PVDF-HFP-based nanocomposite that combines the advantages of capacitive and piezoelectric properties through controling electrospinning environment and processing parameters, polymer solution composition, and addition of nucleating agents such as Carbon Black (CB) to enhance the crystalline development of β-phase, fibre thickness, and morphology. The developed PVDF-HFP/CB nanocomposite presents and response to both slow and fast pressure fluctuations with high capacitance (5.37 nF) and output voltage (1.51 V) allowing for accurate and reliable measurements.

## 1 Introduction

Polymeric sensing materials have emerged as a promising avenue for biomedical applications, as they offer the ability to simultaneously detect and monitor signals or parameters, such as pressure, pH levels, temperature fluctuations, and the presence of specific biomarkers (Zhou et al., 2020; Lin et al., 2023).These sensing materials can be used to develop advanced wearable devices, implantable sensors, and diagnostic tools for various medical conditions. However, further development of responsive capabilities (e.g., sensing range) is needed for practical use in these applications (Guan et al., 2020; Lu et al., 2021). To address these limitations, material design has shifted towards mimicking natural sensing mechanisms and integrating multiple modalities into a single material similar to human skin (He et al., 2020; Lee et al., 2020). Enhancing the sensing capabilities of polymeric materials by emulating the structure and functional behavior of human skin holds promise as potential solutions to this challenge.

The complex sensory function of human skin is governed by SA and FA mechanoreceptors, plays a pivotal role in detecting and responding to tactile stimuli (Knibestöl, 1975). SA mechanoreceptors exhibit a constant response to sustained stimuli, contributing to the perception of static pressure. In contrast, FA mechanoreceptors show a more dynamic response to changing stimuli, contributing to the perception of dynamic pressure (He et al., 2022). Mimicking the function of these mechanoreceptors in sensing materials with both SA and FA characteristics can replicate the responsiveness to slow and fast pressures (Chun et al., 2018; Huynh et al., 2023). Biomimetic sensing materials can also be integrated into wearable devices, diagnostic tools, and as accessories for existing devices such as prosthetics to enhance interaction with the surrounding environment.

PVDF-HFP is a piezoelectric polymer with a high-frequency sensing range that partially mimics the skin’s sensitivity (Lee et al., 2015; Parangusan et al., 2018; Bae and Chang, 2019). These properties are due to the β phase found in PVDF-HFP, which is responsible for its electrical characteristics (e.g., ferroelectric, piezoelectric, and pyroelectric) which have a limited sensing range. Various techniques have been used to enhance this phase, but they are limited by cost and complexity (Bao et al., 2011; Tansel, 2020). Electrospinning has emerged as a promising strategy for inducing stretching and poling effects, enhancing the formation of β phase, and allowing control of morphological features. (Huang et al., 2020; Szewczyk et al., 2020; Yin et al., 2022; Hassan et al., 2023). To build on these capabilities and improve the sensing range it is possible to include conductive fillers like CB can enhance capacitive sensing capabilities even under high strain while maintaining conductive pathways (Zhao et al., 2017; Arduini et al., 2020). Moreover, CB is non-toxic and dispersible in solvents. It is also an inexpensive material with good electrical conductivity. Additionally, its branched nanoaggregates can assemble to form hierarchical structures useful for electrodes, sensors, and biosensors (Brunella et al., 2021; Omar, 2023). Therefore, by incorporating PVDF-HFP with carbon black as a conductive filler via electrospinning, it is possible to create dual sensing polymer nanocomposite with a broad sensing range for both slow and fast pressures.

Here we describe the development of a dual sensing PVDF-HFP/CB nanocomposite sensing material with dual sensing capabilities. The material was developed by controlling the electrospinning environment, polymer composition, and incorporation of nucleating agents. Mimicking the SA and FA mechanoreceptors found in human skin, allows the material to detect both low- and high-frequency movements. Electrical characterisation techniques were used to evaluate the piezoelectric and capacitive sensing properties of the nanocomposite material, confirming its dual sensing capabilities. To explore the material properties contributing to the improved sensing behaviour and range, various characterisation techniques were employed, including scanning electron microscopy (SEM), X-ray diffraction (XRD), Attenuated total reflectance Fourier transform infrared spectroscopy (ATR-FTIR), and *In Situ* SEM analysis of the real-time response of the nanocomposite under tensile tension as a novel method to observe charging/discharging cycles. The optimized composition exhibits capacitance values exceeding 50–100 pF and voltages higher than 300 mV, demonstrating promising performance for applications as a dual-sensing pressure-sensing material.

## 2 Materials and methods

### 2.1 Materials

PVDF-HFP pellets with (Mw ∼ 400,000 kg mol-1), indium tin oxide (ITO) coated polyethylene terephthalate (PET) electrodes with an average area of 20 mm × 10 mm, and polydimethylsiloxane (PDMS) were purchased from Sigma-Aldrich Chemical Co. (United States). CB particles with average diameter of 40 nm was purchased from Alfa Aesar (United States). Solutions were prepared using N, N-dimethylformamide (DMF) and acetone (ACE) both purchased from Sigma Aldrich.

### 2.2 Fabrication of nanocomposite meshes

Homogeneous solution of PVDF-HFP (10%, 15%, 20%, 25%, and 30% w/v) were prepared by using a solvent mixture consisting of DMF and acetone at volume ratio 60/40, which was stirred for 1 h at 40°C. PVDF-HFP/CB (25 wt%), while CB concentrations (2, 4, 6, 8, and 10 wt%.) were prepared following the same procedure but with the additional step of sonicating the CB for 30 min. The mesh sandwiched between the electrodes has been dipped in PDMS for it to be encapsulated by an outer elastic layer for protection. For simplicity, we will refer to the PVDF-HFP meshes as PVDF-HFP10, PVDF-HFP15, PVDF-HFP20, PVDF-HFP25, and PVDF-HFP30. For the PVDF-HFP/CB meshes, it will be referred to as PVDF-HFP/CB2, PVDF-HFP/CB4, PVDF-HFP/CB6, PVDF-HFP/CB8, and PVDF-HFP/CB10.

**FIGURE 1:**
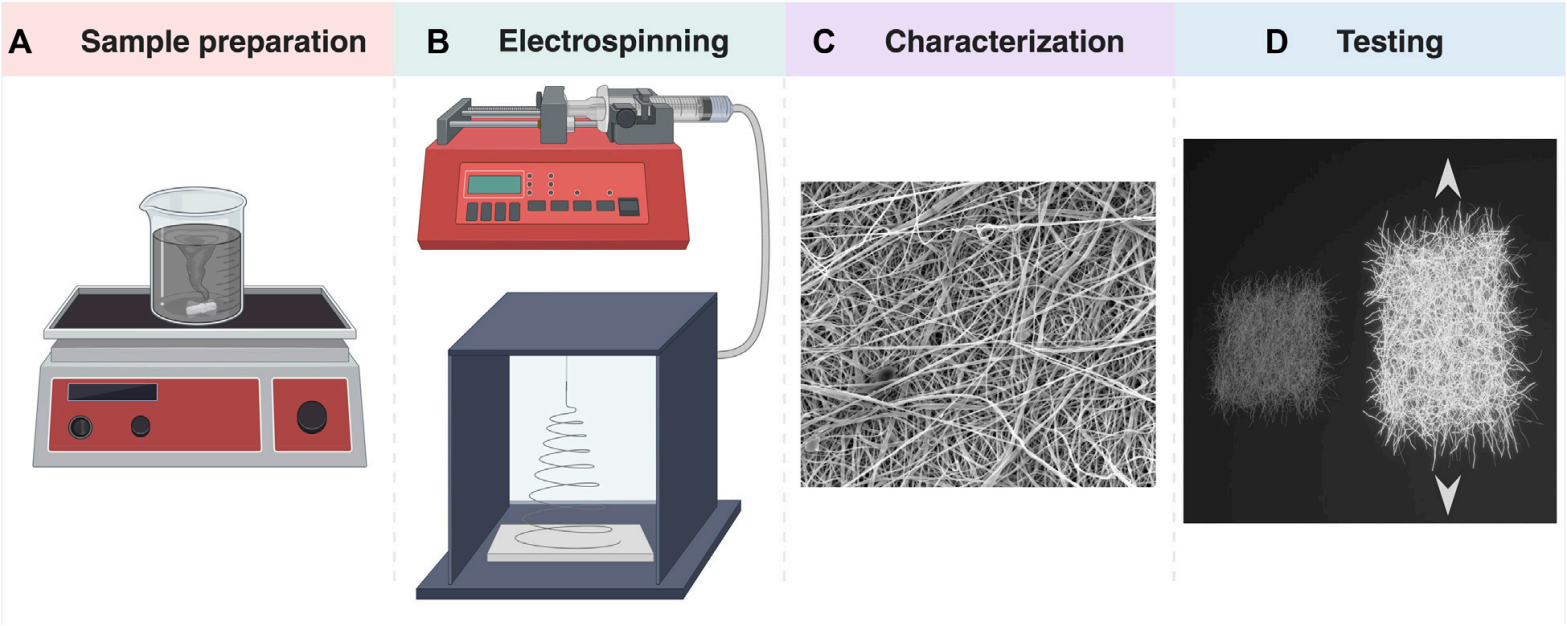
Schematic of the Hybrid sensing material development process: **(A)** Preparation of PVDF-HFP and PVDF-HFP/CB solutions using a magnetic stirrer, **(B)** electrospinning process, **(C)** mesh characterization, and **(D)** *In Situ* testing of meshes.

Meshes were fabricated using a vertical electrospinning system (Prefector, Spraybase, Ireland) equipped with a 0–30 kV power supply, flat collector, and a 20 mL syringe connected using a polytetrafluoroethylene (PTFE) tube to a 18G x 25 mm stainless steel needle (Figure 1). The electrospinning environment during the spinning process was maintained at a relative humidity (RH) of 50% and a temperature of 18°C. Meshes were produced using optimised processing conditions: flow rate 0.8 mL/h, voltage of 14 kV, and tip collector distance (TCD) of 16 cm. The electrospun meshes membranes were sandwiched between ITO coated Polyethylene PET polymer films and then dipped and cured into PDMS.

### 2.3 Morphological characterization

The morphology and structure of the samples was observed using Scanning electron microscopy (SEM) FEI ESEM Quanta 200 system (FEI Company, United States) at 15 kV. The samples were placed on carbon tape fixed onto aluminium stubs and sputter coated using a Cressington 108 coater (Quorom Technologies, UK) with 5 nm platinum (Pt) before imaging. Fibre measurements were carried by taking the average of 50 measurements with ImageJ to identify the size and distribution of nanofibres.

### 2.4 Microstructural characterization

To identify the crystalline structure, Fourier transform infrared (FTIR) spectroscopy in attenuated total reflection (ATR) mode Bruker Alpha II spectrometer with platinum ATR attachment and a diamond crystal was used. Spectra in the 400–1500 cm^−1^ range with a resolution of 1 cm^−1^ was collected to characterize the crystalline structure of the samples. The degree of β crystallinity phase in the samples was calculated as follows (Cai et al., 2017; Xin et al., 2018; Kaspar et al., 2020) the degree was calculated using Eq. 1:

$$F\left(\beta \;\right)=\frac{A_{\beta }}{1.26A_{\alpha }+A_{\beta }}$$ (1)

where Aα and Aβ are the absorbance values at 763 cm^−1^ (CH_2_ in-plane bending or rocking and CF_2_ bending and skeletal bending) and 840 cm^−1^ (CH_2_ rocking and CF_2_ asymmetrical stretching), respectively.

To confirm the crystalline structure, X-ray diffractometer (XRD) X'Pert Pro PANalytical equipped with Cu/Kα radiation (wavelength 0.15418 nm) in the 2θ range of 0°–50° at a scanning speed of 0.1°/min, was used and crystalline content was analysed according to Eq. 2:

$$\frac{I\beta }{\left(I\alpha +I\gamma +I\beta \right)}=\frac{I\left(\frac{200}{110}\right)+I\left(101\right)+I\left(221\right)}{\left(I\left(110\right)+I\left(002\right)+I\left(200/110\right)+I\left(101\right)+I\left(221\right)\right)}$$ (2)

where the Iα, Iγ, and Iβ correspond to the intensity peaks of the α, γ, and β phases, respectively. The XRD peaks for the monoclinic α phase are 18.7° (020), 19.8° (110), and 26.5° (021), while the 20.7° (110/200), 36.6° (101) and 56° (221) peaks correspond to the orthorhombic β phase peak, and the γ phase peak can be attributed to 40° (002) (Cai et al., 2017; Xin et al., 2018; Kaspar et al., 2020):

The average crystallite size (D) was identified using the Scherrer using Eq. 3:

$$D=\frac{K\lambda }{B\cos\theta }$$ (3)

where λ is the wavelength of 1.54 Å, B is the Full Width at Half Maximum (FWHM), θ is Bragg’s and K is a constant 0.94 [54]. The obtained data was analysed using the GraphPad Prism 10 software.

### 2.5 Electrical characterization

The capacitive response was determined using inductance, capacitance, resistance (LCR) Atlas 40 LCR Meter (Peak Electronic Design ltd, UK) while applying a compression static force of 200N using a manual test stand (SAUTER, Switzerland). The piezoelectric responses of the electrospun meshes were measured using a source meter unit (B2901B, Keysight, United States) while performing a finger flexing motion with the sensing material mounted onto a glove.

### 2.6 *In Situ* mechanical deformation

Samples were deformed *In Situ* using a TESCAN and NewTec *In Situ* Tester (TANIST), comprising a NewTec MT1000 (NewTec Scientific, Nimes, France) deformation stage mounted onto a TESCAN Clara (TESCAN Orsay Holding, Brno, Czechia). Free standing electrospun specimens were fixed using a clamping sub fixture within the deformation stage. The deformation stage was configured with a 5 kN load cell. Displacement was recorded using the stages in built linear variable differential transformer (LVDT), with the resistance of the specimen providing negligible force. This approach can be a reliable measure of strain as there is effectively no compliance in the system.

A quasistatic test was performed for each specimen up to −4.5 mm displacement at a rate of 3 μm s^−1^ with a hold every 30 μm to allow for image acquisition and region tracking. The region of interest (ROI) was tracked automatically using template matching based digital image correlation, with a template size of 200 × 200 pixels2. Images were also acquired automatically, with an autofocus function running prior to each imaging increment to maintain the correct work distance. A single field covering the entire ROI was imaged using an Everhart-Thornley detector at 15 kV and 300 pA using a pixel dwell time of 3.2 μs. Such a high voltage is typically a problem for SEM imaging of non-conductive samples but was selected to allow a longer working distance to increase the depth of focus during the experiment to account for the topography of the samples. A small amount of nitrogen was injected (50–100 Pa) into the chamber throughout the experiment to prevent the accumulation of charge due to image acquisition.

## 3 Results

### 3.1 Sensing performance

**FIGURE 2:**
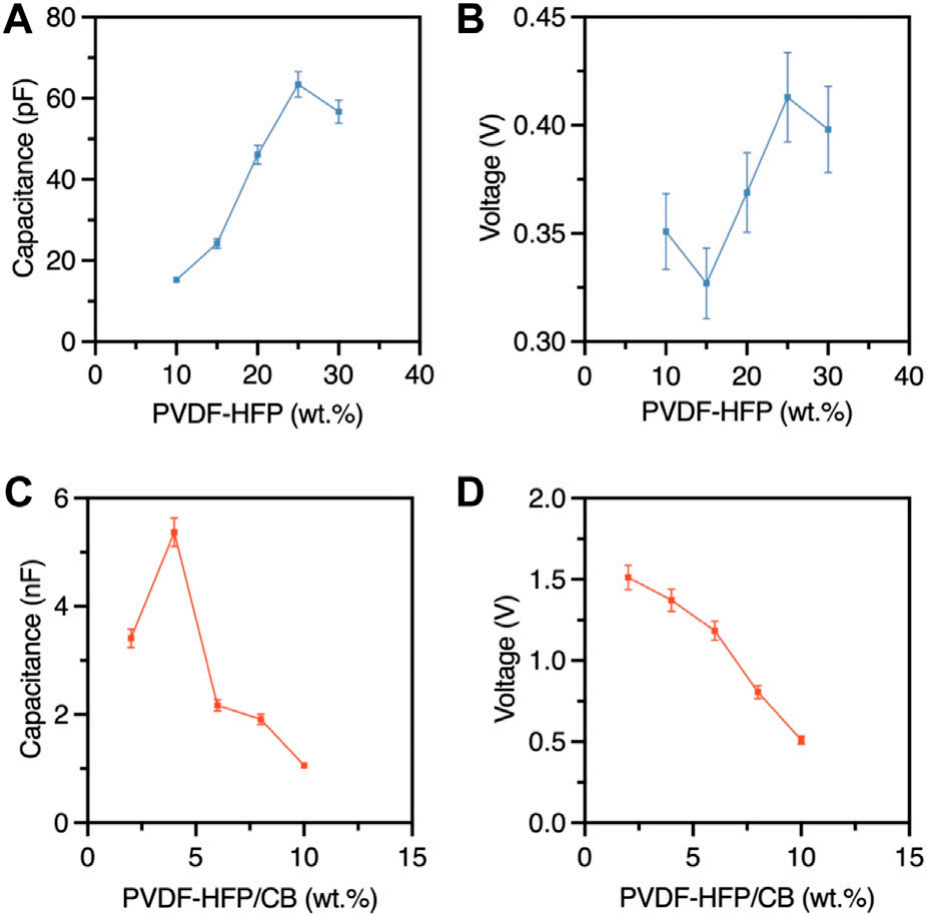
Electrical characteristics of the PVDF-HFP and PVDF-HFP/CB electrospun meshes **(A)** capacitance of PVDF-HFP, **(B)** voltage output of PVDF-HFP **(C)** capacitance of PVDF-HFP/CB, **(D)** voltage output of PVDF-HFP/CB.

The capacitance readings were taken by applying a static force of 200N to the meshes and an average of five capacitance readings were taken (Figures 2A,C). The voltage readings were taken by having the mesh attached to a medical grade glove and an average of five full flexion motions were taken (Figures 2B,D). As observed, the capacitance of the electrospun meshes increases by increasing the concentration of PVDF-HFP up to 25 wt%, after which starts to decrease. Moreover, PVDF-HFP/CB meshes exhibit higher capacitance than PVDF-HFP meshes due the presence of CB, with capacitance increasing by increasing the CB content.

Factors affecting performance and response times can be attributed to the thickness, entanglement, and fibres being in different planes (Li et al., 2023). As observed from SEM images at higher weight percentages of PVDF-HFP and CB the fibres are thicker and more entangled. It can also be observed that the capacitance increases with increasing fibre diameter with PVDF-HFP25 meshes presenting a maximum value of 63.4 pF. However, even though PVDF-HFP/CB2 and PVDF-HFP/CB4 have relatively similar fibre diameters, the capacitance of PVDF-HFP/CB4 is much higher, which can be attributed to the higher CB and β phase contents. Flexion of the finger was performed and an average of five voltage readings were taken (Figures 2A,B). The PVDF-HFP voltage output follows a similar trend to the β phase content. However, the PVDF-HFP/CB2 presents the highest voltage outputs, which decreases by increasing the CB concentration, which can be attributed to the increase in conductivity that negatively affects the piezoelectric effect.

The results highlight the capacitive and piezoelectric properties that the PVDF-HFP and PVDF-HFP/CB meshes possess Supplementary Material S1, S2. The capacitive of the human body ranges from a few pF to 100 pF and the capacitance of the PVDF-HFP meshes is within that range, whereas the PVDF-HFP/CB is much higher indicating a higher sensitivity to the same applied force. Moreover, the output voltage of PVDF-HFP ranges between 0.325 V and 0.425 V and similarly the sensitivity is relatively higher in the case of PVDF-HFP/CB which ranges between 0.5 V and 1.5 V. It is also noted that when testing for these properties the capacitance is sustained during the application of force and was released only afterwards, whereas the output voltage signal spikes during the flexion movement. This exhibits a behaviour like the SA and FA mechanoreceptors found in human skin, where the SA receptors signal remains during sustained stimuli, while FA receptors are adaptive to the changes in stimuli. Mimicking such characteristics is a promising method to have an adaptive dual sensing material that can perceive static and dynamic pressure, texture, and shapes.

### 3.2 Electrospinning nanocomposite polymer meshes

**FIGURE 3:**
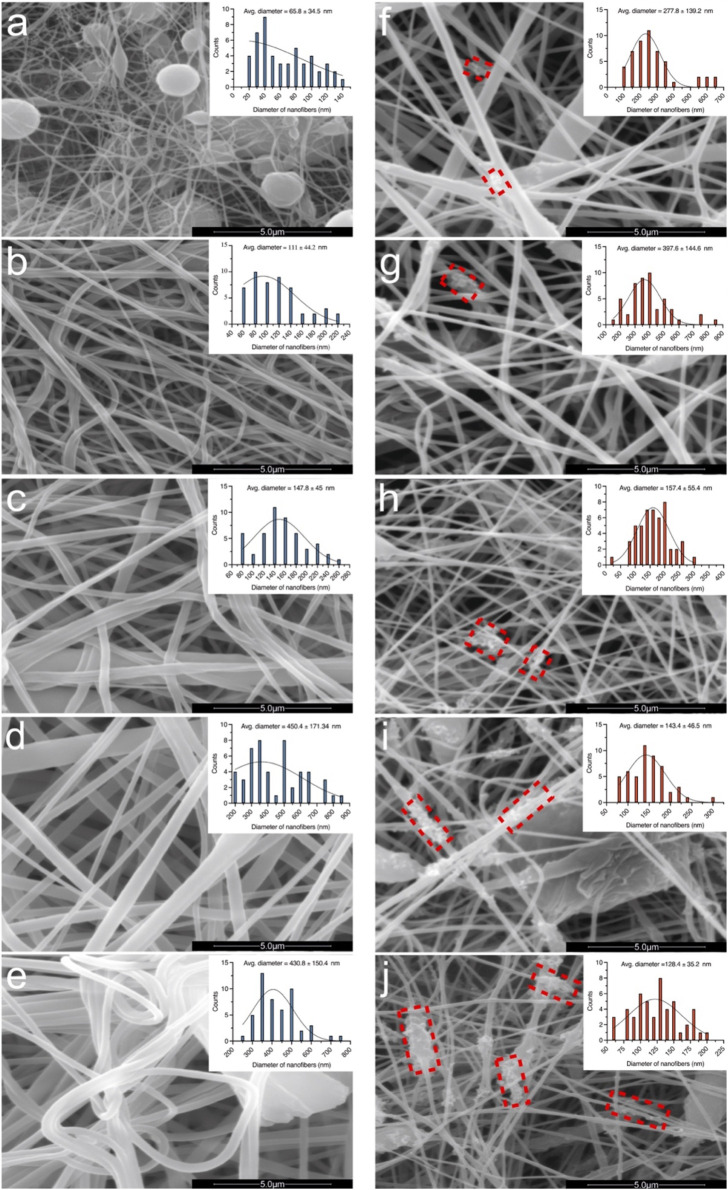
High resolution SEM images and histograms of the measured nanofibres. **(A)** PVDF-HFP10, **(B)** PVDF-HFP15, **(C)** PVDF-HFP20, **(D)** PVDF-HFP25, **(E)** PVDF-HFP30, **(F)** PVDF-HFP/CB2, **(G)** PVDF-HFP/CB4, **(H)** PVDF-HFP/CB6, **(I)** PVDF-HFP/CB8, and **(J)** PVDF-HFP/CB10. The red dashed boxes highlight the CB clusters forming on the nanofibres. Scale bars: 5 μm.

The morphologies of the produced electrospun meshes are presented in Figure 3. As observed, the PVDF-HFP meshes exhibit smoother, more entangled, and on average thicker fibres with increasing polymer concentration. However, the PVDF-HFP/CB meshes present rougher, less entangled, and on average thinner fibres with increasing CB concentration. The measurements of fibre diameters for the PVDF-HFP meshes ranges between 65–450 nm (Figures 3A–E), and for the PVDF-HFP/CB it ranges between 128–398 nm (Figures 3F–J).

A challenge faced during the experimental process was reducing the bead content of the initially fabricated meshes. The electrospinning environment was monitored, and the Relative Humidity (RH) was controlled using humidifiers to identify its effect on the solidification mechanisms and the morphology of PVDF-HFP fibers. The general observation is the higher RH reduces the bead content and produces smoother fibres.

The observed behaviour can be attributed to the different characteristics of the ACE and DMF solvents, creating two stages of jet solidification. The initial solidification occurs at a rapid rate due to the rapid evaporation of the ACE at high RH, thus creating an outer shell. Additionally, the rapid evaporation rate of ACE, can also allow water to polarize PVDF-HFP due to the strong hydrogen bonding between the fluorine atoms and water molecules, thus promoting the formation of β phase (Li et al., 2023). Subsequent solidification occurs at a slower rate due to the higher stability of the DMF in the jets core allowing more time for nucleation while promoting the formation of β phase. As a result, it was found that RH ∼ 50% produced homogeneous fibres, due to changes in the solidification mechanism of the PVDF-HFP fibres (Szewczyk and Stachewicz, 2020). This two stage solidification process induces polarization effects on the surface of the fibre as ACE rapidly evaporates and enhances the nucleation and growth process internally due to the DMF slower evaporation, thus, creating smoother fibres and promoting higher β phase (Zaarour et al., 2018; Mailley et al., 2021).

After reducing the bead content, the effect of polymer concentration was investigated to identify optimal macromolecular entanglements and its effect on the solution’s viscosity. The molecular weight of a polymer or the concentration of a polymer in a solution affects the entanglement density which affects the polymer flows (viscosity). Solutions below the minimum entanglement density will be overstretched due to the low viscosity resulting in thinner fibres, bead formation and breakage of fibres (Wang et al., 2016). Solutions above the maximum entanglement density will be under-stretched due to the high viscosity resulting in thicker fibres, resists fibres alignment, and the growth of β phase (Wang et al., 2016). This was observed for the pure PVDF-HFP and the optimal polymer concentration (25 wt%) allows adequate degree of stretching and an optimal solidification rate producing smooth fibres, higher thickness, and higher β phase.

The addition of CB fillers changes the charge density and conductivity of the solution, and at higher concentrations it can increase the degree of stretching creating thinner fibres (Ma et al., 2011; Cacciotti et al., 2014). Additionally, as the CB content increases it is possible to note the formation of visible clusters on the fibre surfaces (Figures 3F–J). CB being conductivity increases the solution conductivity as well as surface charge density increases. Therefore, as the CB content increases above, so will the electrostatic force generated by the electric field which increases the stretching effect on the jet and resulting in thinner fibres (Figures 3F–J). This can explain the presence of beads at CB contents higher than 4%, which can disrupt formation of β phase. These findings agree with the literature and previous work conducted on polymeric fibres report a percolation limit of 3% and also demonstrate an increased bead content at higher concentrations (Choi et al., 2019; Omar, 2021).Overall, meshes presenting high β phase exhibit smooth surfaces as fibre diameters above 400 nm.

### 3.3 Microstructural development

**FIGURE 4:**
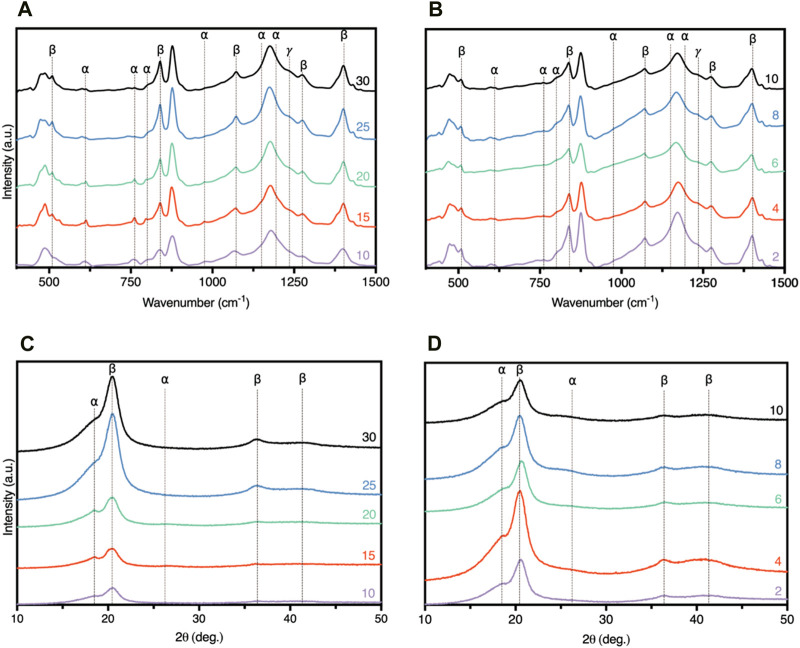
FTIR spectra of **(A)** PVDF-HFP, **(B)** PVDF-HFP/CB, and XRD Spectra of **(C)** PVDF-HFP, and **(D)** PVDF-HFP/CB.

The crystallinity of the PVDF-HFP and PVDF-HFP/CB meshes were analysed using FTIR spectra as shown in Figure 4. The vibrational peaks found in the spectra allows the identification of the α, β, and γ phases in the samples and their content. It is possible to identify peak around 614, 763, 795, 971, 1149, and 1209 cm^−1^ representing the α phase. Whereas other peaks identified at 511, 860, 1071, 1275, and 1431 cm^−1^ and at 1234 cm^−1^ represent the β and γ phases, respectively (Salimi and Yousefi, 2003; Martins et al., 2014; Ting, 2016; Xu et al., 2017). Peaks used to identify the presence of β phase are 840 and 1275 cm^−1^, however, peaks found at 511, 1060, and 1431 cm^−1^ behave similarly and can be used to confirm the presence of β phase. Similarly, the α phase is identified through the 614 and 763 cm^−1^ peaks, and the peaks at 795, 971, 1149, and 1209 cm^−1^. As observed, the increase of PVDF-HFP concentration from 10 to 25 wt% increases the β phase peak intensity and decreases the α phase peak intensity. However, at 30 wt% the β phase peak intensity decreases, while the α phase increases. A similar trend was observed for CB.

The crystallinity of PVDF-HFP and PVDF-HFP/CB meshes were further studied using XRD analysis as shown in Figure 4. Like FTIR, the analysis of the diffraction peaks found in the spectra allow the identification of α, β, and γ phases in the samples and their content. Additionally, using the Scherrer equation it is possible to find the crystallite size to understand the effect of CB. Diffraction peaks at 20.6° (110/200), and 36.2° (101) were used to identify the β phase, and peaks at 19.8° (110) and 40° (002) used to identify the presence of the α and γ phases (Pradhan et al., 2017; Dang, 2018; Kalimuldina et al., 2020). The trend observed in the XRD data agrees with the FTIR data as the increase in the PVDF-HFP concentration from 10 to 25 wt% increases the β diffraction peaks and decreases the intensity of the α diffraction peaks. Additionally, the β phase increases with increase in PVDF-HFP concentration from 10 to 25 wt% but beyond this concentration the β phase peak intensity decreases and the α phase increases. However, there is no trend with the addition of CB, but the highest β phase content was found in PVDF-HFP/CB4.

**FIGURE 5:**
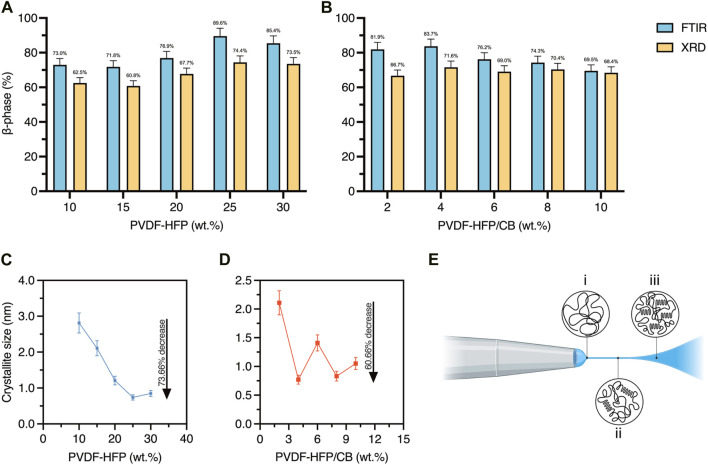
Characterization of the β phase content **(A)** PVDF-HFP and **(B)** PVDF-HFP/CB meshes **(C)** PVDF-HFP crystallite size, **(D)** PVDF-HFP/CB crystallite size, and **(E)** proposed crystal formation during electrospinning (i) amorphous polymer, (ii) formation of lamellar, (iii) increased formation of lamellar stacks.

The results indicate that the higher β phase and lower α phase can be directly correlated with the polymer concentration up to a threshold value (25 wt%). The increase in the polymer concentration reduces the solvent at interface, the vapor pressure, and the evaporation rate (Zhang and Zhang, 2010). Lower evaporation rates of the solvent encourage the growth of β phase as there is more time for highly oriented crystals (lamellar stacks) to form (Figure 5E). Moreover, for the PVDF-HFP/CB meshes, the addition of CB can act as nucleating agent during the electrospinning process.

Additionally, CB has a high surface area and its -OH group can interact with the PVDF-HFP Fluorine atoms to improve polarization and to increase the β phase content (Li et al., 2023). However, the crystallinity improves up to 4 wt%, after which starts to decrease possibly due to CB disrupting the formation of lamellar stacks (Li et al., 2020).

The crystallite size of the β phase in the PVDF-HFP and PVDF-HFP/CB meshes seem to have an inverse relationship with the total β phase content (Figures 5C,D). Moreover, small crystallite sizes seem to be beneficial to the piezoelectric properties, as observed in the PVDF-HFP25 and PVDF-HFP/CB4 meshes, which have the highest β phase percentage (Figures 5A,B) (Yan and Jiang, 2013; Zhou et al., 2018; Parangusan et al., 2019; Jaglan and Uniyal, 2022).

However, at CB concentrations above the percolation limit of PVDF-HFP/CB and a relatively lower crystallinity, it is assumed that the performance would be lower than pure PVDF-HFP meshes. This can be attributed to the crystallite size refinement, as large crystallite sizes produce highly coupled domains which can be polarised easily, thus improving the polarisation effect introduced by CB -OH group and explaining the improved performance of PVDF-HFP/CB meshes (Guan et al., 2010).

### 3.4 Electrospun meshes sensory behaviour

**FIGURE 6:**
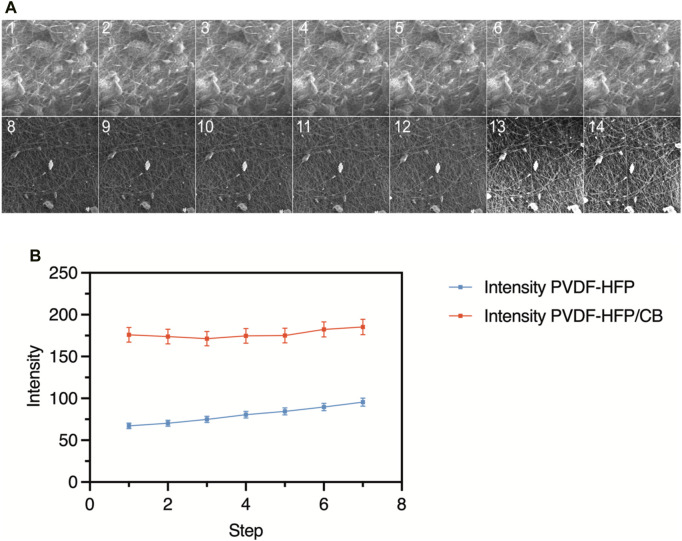
*In Situ* images of PVDF-HFP25 **(A)** (1–7) and PVDF-HFP/CB4 b (8–14), and **(B)** change in brightness during deformation showing the charging and discharging cycles.

Through *In Situ* analysis it was possible to visualize and observe the mesh behaviour during extension of PVDF-HFP/CB4 and PVDF-HFP25 as shown in Figure 6A (1–7) and 6a (8–14), respectively. These two samples were chosen as they present the highest β phase contents. As observed in Figure 6B, there is a notable increase in brightness as the mesh is being stretched, and this charging effect may explain the accumulation of negative ions on the surface of the fibres as observed in previously published work (Li et al., 2020). This accumulation can be due to the activation of the PVDF-HFP during extension, thus increasing negative ions on the surface of the mesh. However, the absolute brightness value is higher for PVDF-HFP/CB4 mesh, and that can be due to the presence of CB filler acting as electron traps. These electron traps also explain the improved PVDF-HFP/CB4 output to that of PVDF-HFP25. Therefore, PVDF-HFP/CB meshes can store more charges for longer periods of time because of these traps, but then result in a slower discharge rate than PVDF-HFP meshes. (Keum et al., 2021). Hence, using *In Situ* SEM it is possible to identify the charging and discharging time, and possibly the time at which the charge is stored during deformation. In this case, it is possible to demonstrate the mechanism of piezoelectric electrospun meshes such as PVDF-HFP and PVDF-HFP/CB during stretching, and the interactions that arise from the addition of fillers.

The results observed from the *In Situ* analysis of PVDF-HFP25 and PVDF-HFP/CB4 during extension can be correlated to the characteristics of SA and FA mechanoreceptors. The increase in brightness during mechanical tension indicates the charging and discharging behaviour. PVDF-HFP/CB4 exhibits a behaviour like SA mechanoreceptors due to the sustained response to mechanical deformation. The electron traps contribute to the slower discharge rate and aligns with the properties of SA mechanoreceptors. On the other hand, PVDF-HFP25 without the presence of electron traps is closely aligned to the characteristics of FA mechanoreceptors due to the shorter response of changes in mechanical deformation. Therefore, the contrasting charging and discharging rates in these meshes are parallel to SA and FA mechanoreceptors emphasizing the potential these meshes in mimicking tactile sensing systems to cater for robotics, prosthetics, and other interactive systems.

## 4 Summary

A simple and low-cost strategy was used to develop PVDF-HFP and PVDF-HFP/CB dual sensing material for biomedical applications. It was hypothesized that PVDF-HFP acts as a dielectric media and the CB as a conductive filler that mimics the electrical output and functionality of SA and FA mechanoreceptors found in human skin. Initial electrical analysis was carried out to identify the performance of the PVDF-HFP and PVDF-HFP/CB sensing materials and prove the dual sensing mechanisms where PVDF-HFP25 and PVDF-HFP/CB4 present highest capacitance (5.37 nF and 63.4 pF) and produced voltage (1.51 V and 0.41 V). This was achieved by controlling the electrospinning environment, polymer composition, and addition of nucleation agents. Using several characterisation techniques it was possible to analyse the morphological and microstructural characteristics using SEM, ATR-FTIR, XRD, and *In Situ* SEM and how attribute to the dual sensing mechanisms. It was observed that PVDF-HFP25 and PVDF-HFP/CB4 electrospun at high RH (−50%) present relatively thicker fibres (−400 nm), smallest crystallite size (0.6 nm), and highest β phase content (−82%). Therefore, it is possible to conclude that fibre thickness, small crystallite sizes, and high β-phase crystallinity both improve and introduce dual sensing mechanisms to the sensing material. This is attributed to the CB -OH group enhancing the polarisation effect, and its use as a nucleating agent to control the microstructural development resulting in higher β-phase and finer crystallite sizes of PVDF-HFP. This study offers a simple and facile method for the fabrication of a dual sensing polymer nanocomposites for biomedical applications that can be used for wearable devices, implantable sensors, and diagnostics tools.

## Data Availability

The original contributions presented in the study are included in the article/Supplementary Material, further inquiries can be directed to the corresponding author.

## References

[B1] Arduini F. Cinti S. Mazzaracchio V. Scognamiglio V. Amine A. Moscone D. (2020). Carbon black as an outstanding and affordable nanomaterial for electrochemical (bio) sensor design. Biosens. Bioelectron. 156, 112033. 10.1016/j.bios.2020.112033 32174547

[B2] Bae J.-H. Chang S.-H. (2019). PVDF-based ferroelectric polymers and dielectric elastomers for sensor and actuator applications: a review. Funct. Compos. Struct. 1 (1), 012003. 10.1088/2631-6331/ab0f48

[B3] Bao S. P. Liang G. D. Tjong S. C. (2011). Effect of mechanical stretching on electrical conductivity and positive temperature coefficient characteristics of poly(vinylidene fluoride)/carbon nanofiber composites prepared by non-solvent precipitation. Carbon 49 (5), 1758–1768. 10.1016/j.carbon.2010.12.062

[B4] Brunella V. Rossatto B. G. Scarano D. Cesano F. (2021). Thermal, morphological, electrical properties and touch-sensor application of conductive carbon black-filled polyamide composites. Nanomaterials 11 (11), 3103. 10.3390/nano11113103 34835866 PMC8619449

[B5] Cacciotti I. Fortunati E. Puglia D. Kenny J. M. Nanni F. (2014). Effect of silver nanoparticles and cellulose nanocrystals on electrospun poly (lactic) acid mats: morphology, thermal properties and mechanical behavior. Carbohydr. Polym. 103, 22–31. 10.1016/j.carbpol.2013.11.052 24528696

[B6] Cai X. Lei T. Sun D. Lin L. (2017). A critical analysis of the α, β and γ phases in poly(vinylidene fluoride) using FTIR. RSC Adv. 7 (25), 15382–15389. 10.1039/c7ra01267e

[B7] Choi H.-J. Kim M. S. Ahn D. Yeo S. Y. Lee S. (2019). Electrical percolation threshold of carbon black in a polymer matrix and its application to antistatic fibre. Sci. Rep. 9 (1), 6338. 10.1038/s41598-019-42495-1 31004091 PMC6474880

[B8] Chun K. Y. Son Y. J. Jeon E. Lee S. (2018). A self‐powered sensor mimicking slow‐and fast‐adapting cutaneous mechanoreceptors. Adv. Mater. 30 (12), 1706299. 10.1002/adma.201706299 29424032

[B9] Dang Z.-M. (2018). Dielectric polymer materials for high-density energy storage. William Andrew.

[B10] Guan F. Wang J. Pan J. Wang Q. Zhu L. (2010). Effects of polymorphism and crystallite size on dipole reorientation in poly(vinylidene fluoride) and its random copolymers. Macromolecules 43 (16), 6739–6748. 10.1021/ma101062j

[B11] Guan X. Wang Z. Zhao W. Huang H. Wang S. Zhang Q. (2020). Flexible piezoresistive sensors with wide-range pressure measurements based on a graded nest-like architecture. ACS Appl. Mater. interfaces 12 (23), 26137–26144. 10.1021/acsami.0c03326 32423195

[B12] Hassan M. H. M. Omar A. Daskalakis E. Grieve B. Bartolo P. J. (2023). Electrospinning polyethylene terephthalate glycol. Int. J. Bioprinting 9, 0024. 10.36922/ijb.0024

[B13] He J. Zhang Y. Zhou R. Meng L. Chen T. Mai W. (2020). Recent advances of wearable and flexible piezoresistivity pressure sensor devices and its future prospects. J. Materiomics 6 (1), 86–101. 10.1016/j.jmat.2020.01.009

[B14] He Z. Ye D. Liu L. Di C. a. Zhu D. (2022). Advances in materials and devices for mimicking sensory adaptation. Mater. Horizons 9 (1), 147–163. 10.1039/d1mh01111a 34542132

[B15] Huang B. Aslan E. Jiang Z. Daskalakis E. Jiao M. Aldalbahi A. (2020). Engineered dual-scale poly (ε-caprolactone) scaffolds using 3D printing and rotational electrospinning for bone tissue regeneration. Addit. Manuf. 36, 101452. 10.1016/j.addma.2020.101452

[B16] Huynh H. Q. Trung T. Q. Bag A. Do T. D. Sultan M. J. Kim M. (2023). Bio‐inspired artificial fast‐adaptive and slow‐adaptive mechanoreceptors with synapse‐like functions. Adv. Funct. Mater. 33 (42), 2303535. 10.1002/adfm.202303535

[B17] Jaglan N. Uniyal P. (2022). On the structural, dielectric, piezoelectric, and energy storage behavior of polyvinylidene fluoride (PVDF) thick film: role of annealing temperature. J. Appl. Phys. 132 (22), 224109. 10.1063/5.0123674

[B18] Kalimuldina G. Turdakyn N. Abay I. Medeubayev A. Nurpeissova A. Adair D. (2020). A review of piezoelectric PVDF film by electrospinning and its applications. Sensors 20 (18), 5214. 10.3390/s20185214 32932744 PMC7570857

[B19] Kaspar P. Sobola D. Částková K. Knápek A. Burda D. Orudzhev F. (2020). Characterization of polyvinylidene fluoride (Pvdf) electrospun fibers doped by carbon flakes. Polymers 12 (12), 2766. 10.3390/polym12122766 33255198 PMC7760733

[B20] Keum K. Heo J. S. Eom J. Lee K. W. Park S. K. Kim Y. H. (2021). Highly sensitive textile-based capacitive pressure sensors using PVDF-HFP/ionic liquid composite films. Sensors 21 (2), 442. 10.3390/s21020442 33435515 PMC7827140

[B21] Knibestöl M. (1975). Stimulus‐response functions of slowly adapting mechanoreceptors in the human glabrous skin area. J. Physiology 245 (1), 63–80. 10.1113/jphysiol.1975.sp010835 PMC1330845 1127614

[B22] Lee J. S. Shin K. Y. Cheong O. J. Kim J. H. Jang J. (2015). Highly sensitive and multifunctional tactile sensor using free-standing ZnO/PVDF thin film with graphene electrodes for pressure and temperature monitoring. Sci. Rep. 5 (1), 7887. 10.1038/srep07887 25601479 PMC4298719

[B23] Lee Y. Park J. Choe A. Cho S. Kim J. Ko H. (2020). Mimicking human and biological skins for multifunctional skin electronics. Adv. Funct. Mater. 30 (20), 1904523. 10.1002/adfm.201904523

[B24] Li H. Mirihanage W. Smith A. D. Donoghue J. Fernando A. (2020). Strain based electrical resistance behaviour of graphene-coated elastomeric yarns. Mater. Lett. 273, 127948. 10.1016/j.matlet.2020.127948

[B25] Li Y. Tong W. Yang J. Wang Z. Wang D. An Q. (2023). Electrode-free piezoelectric nanogenerator based on carbon black/polyvinylidene fluoride–hexafluoropropylene composite achieved via interface polarization effect. Chem. Eng. J. 457, 141356. 10.1016/j.cej.2023.141356

[B26] Lin J.-C. Liatsis P. Alexandridis P. (2023). Flexible and stretchable electrically conductive polymer materials for physical sensing applications. Polym. Rev. 63 (1), 67–126. 10.1080/15583724.2022.2059673

[B27] Lu L. Zhao N. Liu J. Yang B. (2021). Coupling piezoelectric and piezoresistive effects in flexible pressure sensors for human motion detection from zero to high frequency. J. Mater. Chem. C 9 (29), 9309–9318. 10.1039/d1tc01894a

[B28] Ma Q. Mao B. Cebe P. (2011). Chain confinement in electrospun nanocomposites: using thermal analysis to investigate polymer–filler interactions. Polymer 52 (14), 3190–3200. 10.1016/j.polymer.2011.05.015

[B29] Mailley D. Hébraud A. Schlatter G. (2021). A review on the impact of humidity during electrospinning: from the nanofiber structure engineering to the applications. Macromol. Mater. Eng. 306 (7), 2100115. 10.1002/mame.202100115

[B30] Martins P. Lopes A. Lanceros-Mendez S. (2014). Electroactive phases of poly (vinylidene fluoride): determination, processing and applications. Prog. Polym. Sci. 39 (4), 683–706. 10.1016/j.progpolymsci.2013.07.006

[B31] Omar A. M. (2021). “Morphological investigation of electrospun PVDF (HFP)-Carbon black nanocomposites,” in International conference of progress in digital and physical manufacturing (Springer).

[B32] Omar A. M. (2023). “Morphological investigation of electrospun PVDF (HFP)-Carbon black nanocomposites,” in Progress in digital and physical manufacturing (Cham: Springer International Publishing).

[B33] Parangusan H. Ponnamma D. Al-Maadeed M. A. A. (2018). Stretchable electrospun PVDF-HFP/Co-ZnO nanofibers as piezoelectric nanogenerators. Sci. Rep. 8 (1), 754. 10.1038/s41598-017-19082-3 29335498 PMC5768784

[B34] Parangusan H. Ponnamma D. AlMaadeed M. A. A. (2019). Toward high power generating piezoelectric nanofibers: influence of particle size and surface electrostatic interaction of Ce–Fe2O3 and Ce–Co3O4 on PVDF. ACS Omega 4 (4), 6312–6323. 10.1021/acsomega.9b00243 31459771 PMC6648750

[B35] Pradhan S. Kumar A. Sinha A. N. Kour P. Pandey R. Kumar P. (2017). Study of ferroelectric properties on PVDF-PZT nanocomposite. Ferroelectrics 516 (1), 18–27. 10.1080/00150193.2017.1362243

[B36] Salimi A. Yousefi A. (2003). FTIR studies of [beta]-phase crystal formation in stretched PVDF films. Polym. Test. 22, 00003–00005. 10.1016/S0142-9418(03)00003-5

[B37] Szewczyk P. K. Gradys A. Kim S. K. Persano L. Marzec M. Kryshtal A. (2020). Enhanced piezoelectricity of electrospun polyvinylidene fluoride fibers for energy harvesting. ACS Appl. Mater. Interfaces 12 (11), 13575–13583. 10.1021/acsami.0c02578 32090543 PMC7497623

[B38] Szewczyk P. K. Stachewicz U. (2020). The impact of relative humidity on electrospun polymer fibers: from structural changes to fiber morphology. Adv. colloid interface Sci. 286, 102315. 10.1016/j.cis.2020.102315 33197707

[B39] Tansel T. (2020). High beta-phase processing of polyvinylidenefluoride for pyroelectric applications. J. Polym. Res. 27, 95–5. 10.1007/s10965-020-02073-w

[B40] Ting Y. (2016). Design and characterization of one-layer PVDF thin film for a 3D force sensor. Sensors Actuators A Phys. 250, 129–137. 10.1016/j.sna.2016.09.025

[B41] Wang C. Wang Y. Hashimoto T. (2016). Impact of entanglement density on solution electrospinning: a phenomenological model for fiber diameter. Macromolecules 49 (20), 7985–7996. 10.1021/acs.macromol.6b00519

[B42] Xin Y. Zhu J. Sun H. Xu Y. Liu T. Qian C. (2018). A brief review on piezoelectric PVDF nanofibers prepared by electrospinning. Ferroelectrics 526 (1), 140–151. 10.1080/00150193.2018.1456304

[B43] Xu F. Zhang K. Zhou Y. Qu Z. Wang H. Zhang Y. (2017). Facile preparation of highly oriented poly(vinylidene fluoride) uniform films and their ferro- and piezoelectric properties. RSC Adv. 7 (28), 17038–17043. 10.1039/c7ra00586e

[B44] Yan Z. Jiang L. (2013). Size-dependent bending and vibration behaviour of piezoelectric nanobeams due to flexoelectricity. J. Phys. D Appl. Phys. 46 (35), 355502. 10.1088/0022-3727/46/35/355502

[B45] Yin J.-Y. Boaretti C. Lorenzetti A. Martucci A. Roso M. Modesti M. (2022). Effects of solvent and electrospinning parameters on the morphology and piezoelectric properties of PVDF nanofibrous membrane. Nanomaterials 12 (6), 962. 10.3390/nano12060962 35335774 PMC8954422

[B46] Zaarour B. Zhu L. Huang C. Jin X. (2018). Controlling the secondary surface morphology of electrospun PVDF nanofibers by regulating the solvent and relative humidity. Nanoscale Res. Lett. 13 (1), 285–311. 10.1186/s11671-018-2705-0 30209633 PMC6135735

[B47] Zhang H. D. a.J. Zhang J. (2010). Solvent induced shape recovery of shape memorypolymer based on chemically cross-linked poly(vinyl alcohol). Soft Matter 6 (14), 3370–3376. 10.1039/b922220k

[B48] Zhao S. Li J. Cao D. Zhang G. Li J. Li K. (2017). Recent advancements in flexible and stretchable electrodes for electromechanical sensors: strategies, materials, and features. ACS Appl. Mater. interfaces 9 (14), 12147–12164. 10.1021/acsami.6b13800 28281337

[B49] Zhou B. Li R. Cai J. Xu J. Zhao Z. Pei J. (2018). Grain size effect on electric properties of novel BaTiO3/PVDF composite piezoelectric ceramics. Mater. Res. Express 5 (9), 095510. 10.1088/2053-1591/aad8a4

[B50] Zhou N. Liu T. Wen B. Gong C. Wei G. Su Z. (2020). Recent advances in the construction of flexible sensors for biomedical applications. Biotechnol. J. 15 (12), 2000094. 10.1002/biot.202000094 32744777

